# Shortage of Cellular ATP as a Cause of Diseases and Strategies to Enhance ATP

**DOI:** 10.3389/fphar.2019.00098

**Published:** 2019-02-19

**Authors:** Todd A. Johnson, H. A. Jinnah, Naoyuki Kamatani

**Affiliations:** ^1^StaGen Co., Ltd., Tokyo, Japan; ^2^Departments of Neurology and Human Genetics, Emory University School of Medicine, Atlanta, GA, United States

**Keywords:** adenosine triphosphate, cellular energetics, hypoxanthine (PubChem COD: 790), inosine (PubChem CID: 6021), purines, xanthine oxidoreductase inhibitors, cardiovascular diseases, CNS diseases

## Abstract

Germline mutations in cellular-energy associated genes have been shown to lead to various monogenic disorders. Notably, mitochondrial disorders often impact skeletal muscle, brain, liver, heart, and kidneys, which are the body’s top energy-consuming organs. However, energy-related dysfunctions have not been widely seen as causes of common diseases, although evidence points to such a link for certain disorders. During acute energy consumption, like extreme exercise, cells increase the favorability of the adenylate kinase reaction 2-ADP -> ATP+AMP by AMP deaminase degrading AMP to IMP, which further degrades to inosine and then to purines hypoxanthine -> xanthine -> urate. Thus, increased blood urate levels may act as a barometer of extreme energy consumption. AMP deaminase deficient subjects experience some negative effects like decreased muscle power output, but also positive effects such as decreased diabetes and improved prognosis for chronic heart failure patients. That may reflect decreased energy consumption from maintaining the pool of IMP for salvage to AMP and then ATP, since *de novo* IMP synthesis requires burning seven ATPs. Similarly, beneficial effects have been seen in heart, skeletal muscle, or brain after treatment with allopurinol or febuxostat to inhibit xanthine oxidoreductase, which catalyzes hypoxanthine -> xanthine and xanthine -> urate reactions. Some disorders of those organs may reflect dysfunction in energy-consumption/production, and the observed beneficial effects related to reinforcement of ATP re-synthesis due to increased hypoxanthine levels in the blood and tissues. Recent clinical studies indicated that treatment with xanthine oxidoreductase inhibitors plus inosine had the strongest impact for increasing the pool of salvageable purines and leading to increased ATP levels in humans, thereby suggesting that this combination is more beneficial than a xanthine oxidoreductase inhibitor alone to treat disorders with ATP deficiency.

## Introduction

In society and industry, three categories are important: things, information, and energy. Similarly, those three should also be important in biology at the cellular level. Since many diseases are caused by cellular shortages of chemicals (things) and DNA (information), there should also be diseases caused by a shortage of cellular ATP (energy). Based on the June 2018 Integrated Mitochondrial Protein Index from MitoMiner (Smith and Robinson, 2016), over 1,600 proteins coded by chromosomal genes are known to be associated with mitochondria. Since humans have about 20,000 genes (Harrow et al., 2012), that means that about 8% of all genes are associated with mitochondria and with energy. It is of interest that at a societal scale, about 8% of world-wide economic activity is also energy-related (Institute for Energy Research [IER], 2010).

Mitochondria and the glycolytic pathway are responsible for cellular energy production, and germline mutations in the genes associated with them have been shown to lead to various monogenic disorders. Of note, mitochondrial disorders often result in dysfunctions or symptoms involving one or more of the top energy consuming organs (Wallace, 1999), which are skeletal muscle, brain, liver, heart, and kidneys (349.7, 316.8, 278, 136.4, and 129 kcal/day, respectively) (Wang et al., 2010). Especially, heart, skeletal muscle, and the brain are often observed as targets of mitochondrial disorders, but dysfunction has also been observed in the other high-energy utilizing organs such as the liver (Lee and Sokol, 2007) and kidneys (Rahman and Hall, 2013; Che et al., 2014; Emma et al., 2016; Emma and Salviati, 2017). Additionally, mutations in genes related to mitochondrial function sometimes lead to pathology in other small organs/tissues that utilize large amounts of energy relative to their size or weight such as inner-ear hair cells (Hinson et al., 2007; Tiede et al., 2009; Yan et al., 2017) or structures of the eye such as retina, optic nerves, and extraocular muscles (Barot et al., 2011; Schrier and Falk, 2011).

Although rare genetic diseases such as mitochondrial diseases are known to be associated with energy, for common diseases, energy shortage has not been studied as extensively as other potential causes. For example, titles/abstracts in PubMed that mentioned etiology/causes and Alzheimer’s disease (AD; *n* = 6318), Parkinson’s disease (PD; *n* = 4602), heart disease (heart or cardiac disease/failure, heart; *n* = 20643), or diabetes (*n* = 22698) only included energy related terms (cellular energy, energetics, bioenergetics, energy and mitochondria, mitochondrial function, mitochondrial dysfunction) for a small percentage of papers (AD = 3.7%, PD = 7.9%, heart = 0.6%, diabetes = 1.3%). Notably, for years, amyloid, tau, and alpha-synuclein hypotheses have dominated AD and PD research (Zhang et al., 1989; Stefanis, 2012; Ozansoy and Başak, 2013), but recently, researchers have suggested that mitochondrial or bioenergetic dysfunction may be related to etiology of AD or PD (Winklhofer and Haass, 2010; Wellstead and Cloutier, 2011; Desler et al., 2017; Onyango et al., 2017; Swerdlow et al., 2017).

## Cellular Energy-Charge and ATP Turnover

**FIGURE 1:**
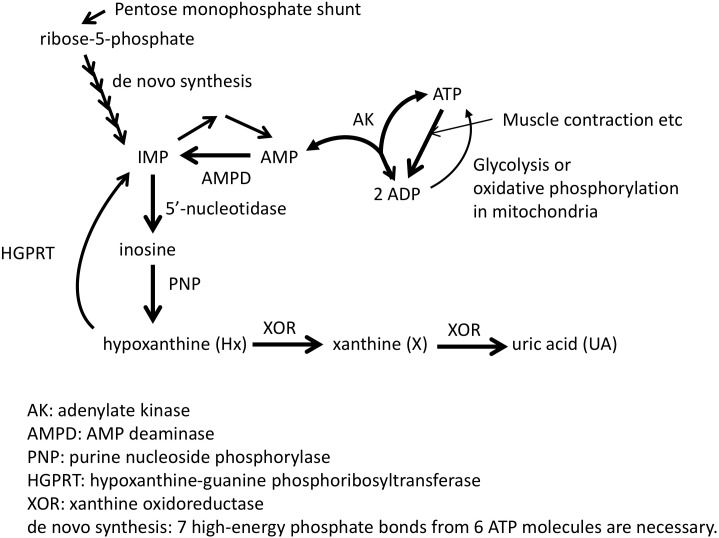
Pathway related to ATP synthesis and degradation. Adapted from Kamatani et al. (2017) with permission of the journal.

Adenosine triphosphate (ATP) is known as the “energy currency” of the cell, and central to use of that currency is the system’s ability to generate and maintain levels of what is known as the “energy charge,” the ratio of the concentrations [ATP+0.5^∗^ADP]/[ATP+ADP+AMP] (Chapman and Atkinson, 1973). Although mitochondrial and glycolytic pathways are used to produce energy from molecules such as sugars, proteins, and fatty acids, instantaneous energy needs are satisfied first through the phosphocreatine (PCr) shuttle (Guimarães-Ferreira, 2014) and then through the combined efforts of AMP deaminase (AMPD), AMP-activated protein kinase (AMPK), and adenylate kinase (AK) (Panayiotou et al., 2014). AMPK acts as a form of energy charge sensor (Hardie et al., 2016), which regulates AMPD activity, while AMPD deaminates AMP to IMP to maintain higher values of the energy charge (Lanaspa et al., 2012; Plaideau et al., 2014; Lanaspa et al., 2015) and favor the forward AK reaction that produces ATP and AMP from two ADP molecules (Figure 1; Saks et al., 2014). IMP may then be degraded to inosine via 5′-nucleotidase and then to hypoxanthine (Hx) by purine nucleotide phosphorylase (PNP) and potentially further degraded to xanthine (X) and uric acid (UA) through xanthine oxidoreductase (XOR) (Figure 1; Maiuolo et al., 2016). Thus, such purine molecules form the scaffold of the key molecule for storing cellular energy.

### Considering Differences Between Humans and Animal Models

When comparing and interpreting results from studies based on animal-models versus those from human subjects, researchers should consider both differences in metabolic rates and biochemical pathways that exist between species. While safety is of paramount importance, not accounting for such differences may also potentially lead one not to consider developing a drug based on phenomena observed in animal models that do not apply to humans.

One notable difference relates to Kleiber’s Law, which states that an organism’s resting energy expenditure (REE) relates to its mass (M) as *REE∝M*^3/4^ (Kleiber, 1932; Brody, 1945). Thus, *REE* per-unit-mass (*REE*/*M*) for small animals is much greater than that of large animals; *REE*/*M* is about eight times greater in mice (196 kcal/kg per day) than in humans (24.8 kcal/kg per day) (Wang et al., 2012). Commensurate with a smaller animal’s need to generate energy and therefore resynthesize ATP at a much faster rate, other studies found that excretion of purine degradation products was about seven-times higher in dogs and 40-times higher in rats than in humans (Hitchings, 1966).

Another notable difference in purine metabolism relates to the different end-products of purine degradation. Guanosine and adenosine nucleotide degradation pathways converge on X, which is then degraded in most mammals to slightly soluble UA by XOR and readily soluble allantoin by urate oxidase (UOX) (Johnson et al., 2009). However, a string of *UOX* genetic mutations in the ape-lineage finally led to its pseudogenization in humans (Kratzer et al., 2014), meaning that the terminal end-product of purine degradation is different in humans (UA) and typical animal models (allantoin). At least partly due to their increased metabolic rate, UOX knockout in mice results in extreme hyperuricemia and is highly lethal (Wu et al., 1994), while it is generally benign in humans (who are all *UOX* knockouts). In real-world terms, this is an example of the importance of considering human/animal-model differences, because drugs used to treat hyperuricemia such as allopurinol and febuxostat resulted in renal calculi formation in mice and rats but were not expected to cause such problems in humans (Horiuchi et al., 1999).

Taken together, these observations and results suggest that both similarities and differences between biochemical and metabolic pathways need to be considered when using animal models to extrapolate about biologic and clinical phenomena in humans.

### Xanthine Oxidoreductase (XOR): A Key Enzyme in Purine Salvage

Xanthine oxidoreductase is a homodimeric metallo-flavoprotein that has in its N-terminal region two iron-sulfur redox centers, at its C-terminal domain a catalytic center with the molybdenum cofactor molybdopterin (Moco), and the intervening region a flavin-adenine dinucleotide (FAD) binding domain (Battelli et al., 2014). Research almost 100 years ago measured XOR activity in cow milk, and showed in various rat tissues that it had highest activity in liver and spleen, intermediate activity in kidney and lungs, and little to no activity in muscle (Morgan et al., 1922; Massey and Harris, 1997).

#### Localization of XOR in Human Tissues, Cell-Types, and Blood

**FIGURE 2:**
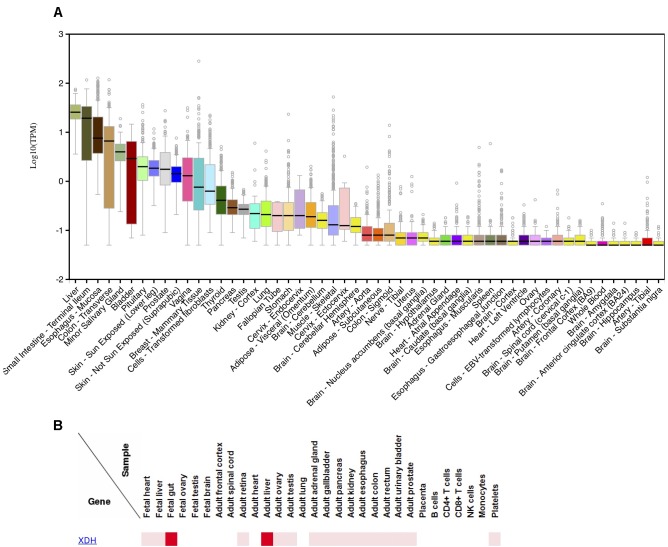
Expression of XDH in various human tissues.**(A)** Distribution of log10 (TPM) of XDH RNA-seq expression levels in 53 human tissues; produced 9/25/2018 on the GTEx Portal^5^. **(B)** Relative XDH protein expression levels in human tissues and cell-lines; produced 10/11/2018 on the Human Proteome Map web-site^6^.

Several lines of evidence can be used to understand the presence/absence of XOR in various human tissues and cell types. First, we examined results from several human immunohistochemistry (IHC) studies that investigated XOR protein localization using anti-XOR antibodies (Abs) (Hellsten-Westing, 1993; Moriwaki et al., 1996; Linder et al., 1999, 2005; Martin et al., 2004). Second, we analyzed gene expression data for *XDH* from the GTEx Portal browser^1^ (Supplementary Table S1; *n* = 53; GTEx Consortium, 2017) and the FANTOM5 human Phase 1and 2 promoterome^2^ (Supplementary Table S2; *n* = 1829; Hon et al., 2017; Lizio et al., 2015, 2017), and for comparison with a commonly analyzed model species, for mouse *Xdh* data from the Comprehensive Mouse Transcriptomic BodyMap^3^ (Supplementary Table S3; *n* = 17; Li et al., 2017) and the FANTOM5 mouse promoterome^4^ (Supplementary Table S4; *n* = 1195; Abugessaisa et al., 2017; Noguchi et al., 2017). Examination of the human and mouse FANTOM5 datasets, which assayed large numbers of tissues and cell-types, suggested that expression within a species can vary by tissue sub-location and also across cell-types within a tissue. Finally, data from the Human Proteome Map provides additional evidence for XOR protein in various tissues (Kim et al., 2014). Human tissue expression levels from GTEx Portal RNA-seq data (Figure 2A) closely mirrored those observed in protein expression data from the Human Proteome Map (Figure 2B; Kim et al., 2014).

Among multi-tissue IHC analyses, Linder et al. (1999) found the highest XOR staining in liver periportal hepatocytes and Kupffer cells, various proximal intestine enterocytes and goblet cells, and lactating mammary gland ductal epithelium. FANTOM5 data agreed with those findings, with liver, small-intestine/intestine, and mammary gland tissues or cell-types among high expressing samples in both human and mouse adult datasets. While small-intestine was top ranked in human FANTOM5, in the mouse, intestinal mucosa had six times the relative expression levels compared to whole adult intestine, suggesting that expression is greater in certain intestinal cell subsets. Moriwaki et al. (1996) found moderate to high levels of XOR in epithelium from various tissues including small and large intestine, trachea, bile duct, esophagus, tongue, breast mammary tissue, and lung bronchi. In human FANTOM5, 41% of human epithelial samples (30/73) expressed *XDH*, including a majority of bronchial (4/7) and all small-airway (3/3) epithelial samples.

Both Moriwaki et al. (1996) and Linder et al. (1999) found that brain tissues were negative for XOR expression. In agreement, FANTOM5 human data showed no *XDH* expression in human brain samples (brain, adult samples: 0/2; Astrocyte-cerebellum: 0/3; Astrocyte-cerebral cortex: 0/3; cerebellum, adult 0/3; cerebral meninges: 0/1; hippocampus: 0/4; substantia nigra: 0/4), and all thirteen of GTEx Portal brain tissues had median TPM values of 0 or if expressed, were less than 0.5% that of liver.

In skeletal muscle, Hellsten-Westing (1993) and Linder et al. (1999) did not detect XOR in myocytes, and in human gene expression data, human skeletal muscle samples displayed no or low *XDH* expression, with no expression in adult FANTOM5 samples (0/6) and GTEx samples having a very low median TPM of 0.08 (0.31% of liver). In contrast to human data, the FANTOM5 mouse biceps femoris muscle sample showed strong expression, with a relative-log expression (RLE) of 52.0 (19.6% of that in liver). In heart, Linder et al. (1999) found no staining for XOR across all cell-types, Hellsten-Westing (1993) found no staining of myocytes, while Moriwaki et al. (1996) reported weak staining of cardiac myocytes. Both GTEx heart tissues (Atrial appendage; Left ventricle) showed a very low median TPM of 0.01 (0.04% of liver), and FANTOM5 human heart samples and cardiac-related cell-types showed no *XDH* expression (heart, adult diseased: 0/2; heart, adult, pool: 0/1; heart, fetal, pool: 0/1; Cardiac myocytes: 0/3; Fibroblast-Cardiac: 0/6; mesenchymal precursor cell-cardiac: 0/4). In contrast, mouse BodyMap heart tissue and FANTOM5 cardiac myocytes displayed high *Xdh* expression (BodyMap: mean FPKM 18.47 or 18.8% of small intestine; FANTOM5: 45.6-181.4 RLE or 17–68% of liver).

In both skeletal muscle and heart, Hellsten-Westing (1993) identified capillary endothelium as weakly positive and smooth muscle cells of arterioles and venules as strongly positive for XOR, respectively. Linder et al. (1999) and Moriwaki et al. (1996) also made the general observation that XOR stained capillary endothelium, but Linder et al. (1999) did not observe staining of heart blood vessels, and the authors pointed to a lack of knowledge as to whether vascular endothelial cells expressed *XDH* mRNA, or rather, if XOR from the blood was bound to sulfated glycosaminoglycans on the endothelial cell membrane (Adachi et al., 1993; Radi et al., 1997). To answer that question, we examined mouse and human FANTOM5, and while the mouse data provided some support for localized XOR expression, with the single mouse endothelial sample (hepatic Sinusoidal Endothelial Cells) displaying very strong *Xdh* levels (89.6% of liver), no human vascular endothelial cell samples expressed *XDH* (Aortic:0/3; Artery 0/3; Microvascular 0/3; Vein 0/3; Umbilical vein: 0/3; Vein: 0/3). Similarly, *Xdh* was strongly expressed in the single mouse aortic smooth muscle cells sample, but human vascular smooth muscle samples overall lacked *XDH* expression (Aortic: 1/4; Brachiocephalic: 0/3; Brain vascular 0/3; Carotid: 0/3; Coronary artery: 0/3; Pulmonary artery: 0/3; Subclavian artery: 0/3; Umbilical artery: 0/4; Umbilical vein: 0/3).

Similar to our findings for endothelial and vascular smooth muscle cells, previous reports described XOR as a regulator of adipose tissue differentiation and found that mouse white adipose tissue (WAT) had ∼30% higher expression levels compared to liver (Cheung et al., 2007; Murakami et al., 2014). In contrast, GTEx human subcutaneous and visceral fat tissue had low median expression of 0.03 TPM and 0.14 TPM, respectively, (0.12 and 0.55%, of liver) and none of FANTOM5 adult adipose tissue samples or mesenchymal adipose related cells (precursors, stem cells, and during adipogenic induction) showed detectable *XDH* expression.

#### Circulating XOR as a Source of Endothelial Protein-Binding/Activity

Since liver is one of the top sites of XOR activity in humans, damage to the liver has been suspected to allow XOR to enter the circulation (Tan et al., 1993; Martin et al., 2004). In tests of hepatotoxic agents in animal models, it was shown that XOR is released into the blood (Giler et al., 1976, 1977; Zima et al., 1993; Zima et al., 2001). Additionally, since ischemia-reperfusion (I-R) can result in injury to organs, researchers investigated I-R induced in animal models as well as I-R in human subjects undergoing surgery. In rabbits, occlusion of the thoracic aorta resulted in four-fold increases in plasma levels of liver enzymes (AST, LDH) and XOR during post-ischemia reperfusion, suggesting that ischemia caused liver damage and leakage of various cellular enzymes, including active XOR, accompanied restored blood flow (Nielsen et al., 1994). In similar human surgeries, circulating XOR was increased (∼two-fold increase), likely from damaged liver and intestine due to the aortic cross-clamp procedure, and was suggested as a source of ROS activity that could cause damage in downstream organs in the circulation that normally lack XOR expression such as the heart or lungs (Tan et al., 1995).

However, within normal human plasma, XOR levels have historically been described as undetectable or very-low (Fried and Fried, 1974). Using a new more sensitive assay (Murase et al., 2016), a recent study found XOR activity in plasma from 282 Japanese subjects of 32 (19–58) pmol/h/ml (median and interquartile range) (Furuhashi et al., 2018). However, our calculations show that plasma XOR at those concentrations would only account for a negligible amount: about 0.09% of daily production [(32 × 10^-12^ moles/h/ml^∗^5000 ml^∗^24 h/day^∗^168.1103 g/mole)/0.7 g/day]. Thus, under certain pathologic conditions, XOR levels may substantially increase and lead to downstream effects (Battelli et al., 2014), but under normal physiologic conditions, the amount of circulating XOR is notably low and unlikely to elicit local pathology.

#### Xanthine Dehydrogenase Is the Predominant Form of XOR

Depending on the species, the XOR enzyme may degrade Hx to X through either xanthine dehydrogenase (XDH) or xanthine oxidase (XO) enzymatic activities (Stirpe and Della Corte, 1969):

XDH:

$$\mathrm{Hx}+H_{2}O+{\mathrm{NAD}}^{+}\to X+\mathrm{NADH}+H^{+}$$

$$X+H_{2}O+{\mathrm{NAD}}^{+}\to \mathrm{UA}+\mathrm{NADH}+H^{+}$$

XO:

$$\mathrm{Hx}+H_{2}O+O_{2}\to X+H_{2}O_{2}$$

$$X+H_{2}O+O_{2}\to \mathrm{UA}+H_{2}O_{2}$$

The mechanism of transition between XDH and XO activities has been described as dependent on either proteolytic nicking or disulfide bonds formed between cysteine residues in two parts of the enzyme structure (amino acids residues: Cys535-Cys992 and the C-terminal Cys1316-Cys1324) (Della Corte and Stirpe, 1968; Corte and Stirpe, 1972). Oxidation of Cys535-Cys992 to form a disulfide bond occurs at a fast pace, while that of the C-terminal residues occurs much more slowly (Nishino et al., 2015). A clearer picture of the conversion mechanism was identified by Nishino and collegues using X-ray crystallography of modified versions of the enzyme that lock one or the other activities in place (Nishino et al., 2008b) or that removed the C-terminal peptide of the enzyme (Nishino and Okamoto, 2015). Those experiments showed that the C-terminal peptide in the structure undergoes insertion into the FAD cavity, and that that conformation maintains XDH activity. From that, it was proposed that interactions with cell membranes, such as in capillary endothelium, by the C-terminal peptide may facilitate conversion to the XO form. Although the analyses of various O_2_ levels and cultured human bronchial epithelial cells showed that XOR activity increased with decreasing O_2_ levels, XO as a percent of total XOR activity (XO/XOR) was reported to remain unchanged, and XO/XOR was about 20% at each oxygen level (Linder et al., 2003).

Early studies that examined XDH to XO conversion did so using tissue homogenates (Corte and Stirpe, 1968; Stirpe and Della Corte, 1969; Engerson et al., 1987; McKelvey et al., 1988), and one of the more comprehensive reports found that in rat lung, kidney, liver, and heart, XO/XOR was between 10 and 40% if homogenates were processed and assayed quickly (Engerson et al., 1987). However, it was noted that prolonged time in culture, especially under conditions simulating ischemia, resulted in XO/XOR between 50 and 100%. In human normal liver homogenates, XO/XOR of about 20–25% was observed, while in patients with liver cirrhosis, it increased to between 35 and 45% (Stirpe et al., 2002).

Since the XO/XOR ratio increased with longer incubation times of the tissue homogenate, one study tried to obtain true *in situ* values by examining XDH and XO activities via histochemical analysis of rat liver sections and found that XO activity only represented about 4% of total activity, which suggested that XDH represented the physiologically predominant XOR form (Frederiks and Bosch, 1996). In addition, they found that under ischemic conditions, XO/XOR did not increase, but rather, that it slightly decreased (Frederiks et al., 1993; Frederiks and Bosch, 1996). To understand whether a “xanthine oxidase activating factor” was present in homogenized versus intact tissue, another report found that freeze-thaw cycling of tissue samples released a mitochondrial protease that caused irreversible XO conversion (Saksela et al., 1999). Taken together, such past reports suggest that XDH represents the physiologically normal activity of XOR, even under certain pathologic conditions.

### Purine Salvage and the “Circulating Hypoxanthine Pool”

Purine molecules form the scaffold for the high-energy phosphates such as ATP and GTP, and they can be either produced *de novo* or salvaged from nucleobases or nucleosides that have either been previously synthesized and undergone partial degradation or obtained from the diet (Ipata et al., 2011). However, since *de novo* synthesis expends energy in the form of 7 high-energy phosphate bonds from 6 ATP molecules to regenerate IMP (Figure 1; Garrett and Grisham, 2016), energy-intensive tissues such as skeletal muscle, heart muscle cells, and brain neurons extensively use salvage pathways to maintain their purine levels (Manfredi and Holmes, 1985; Ipata, 2011). In that process, three purine nucleobases can be salvaged by two different enzymes, with adenine phosphoribosyltransferase (APRT) converting adenine (Ade) to AMP, or hypoxanthine-guanine phosphoribosyltransferase (HGPRT; gene *HPRT1*) converting Guanine (Gua) and Hx to GMP and IMP, respectively. After the salvage of Hx, IMP may then be converted by Adenylosuccinate synthetase (ADSS; genes *ADSS* and *ADSSL1*) to S-AMP (adenylosuccinate) which is then converted by Adenylosuccinate lyase (ADSL) to AMP.

That process of purine salvage and its importance was proposed over 40 years ago, and analyses at the time showed that a substantial fraction (∼95%) of the body’s production and intake of purines is salvaged and recycled from Hx back into nucleotides (Murray et al., 1970; Murray, 1971). Analyses of purine metabolism showed that plasma and urine purine levels varied with different levels of physical activity, and Harkness et al. (1983) observed that acute muscular exercise resulted in dramatically increased plasma Hx levels, and from that, they proposed that the release of Hx and its transport between tissues could be considered as a “circulating hypoxanthine pool” (Harkness et al., 1983). Later, analyses indicated that the total purine loss (i.e., excreted in 24 h urine samples) increased with the number of severe acute exercise sessions (Stathis et al., 1999), and Hellsten et al. (1998) showed that after intense exercise, Hx and UA in the blood increased during recovery, and that while salvage pathways recovered IMP still present in the muscle into ATP, Hx that had been released into the blood appeared not to be salvaged, but rather, Hx was degraded into UA by the liver, returned to the circulation, and then taken up by the muscle tissue. A later study by Stathis et al. (2005) investigated whether treatment with an XOR inhibitor (allopurinol) would attenuate total purine output and allow for greater Hx salvage by the muscle after intense exercise, but allopurinol administration was found to actually increase total purine loss. That study also illustrated that Ino and its metabolite Hx can be readily transported out of muscle cells once IMP is de-phosphorylated by 5′-nucleotidase, and that the increased concentrations of Ino and Hx in the blood may then be readily removed into the urine by the kidneys. The high-transfer potential of Ino and Hx out of exercised muscle likely reflects the action of equilibrative nucleoside transporters ENT1 (*SLC29A1*) and ENT2 (*SLC29A2*) that passively move nucleosides and nucleobases out of cellular compartments with high metabolite concentrations (Boswell-Casteel and Hays, 2017).

Much of our early knowledge on the existence of purine salvage originated from research on patients with Lesch-Nyhan’s Disease (LND), which results in “mental retardation, spastic cerebral palsy, choreoathetosis, uric acid urinary stones, and self-destructive biting of fingers and lips” (Hamosh, 2017). LND is caused by genetic mutations in the *HPRT1* gene that result in a deficiency in HGPRT enzyme function (Seegmiller et al., 1967; Nguyen and Nyhan, 2016). Thus, LND patients are not able to salvage Gua and Hx to GMP and IMP, respectively, and they display increased levels of purine metabolites including Hx, UA, X, and Ino in the blood (Jiménez et al., 1989a). Analysis of the distribution of purine metabolites in the plasma and urine through use of radioactive tracers suggested that purine salvage was a normal mechanism for Hx reutilization in humans (Edwards et al., 1979), and other researchers showed that purines produced in the liver were taken up by erythrocytes and transported to other tissues (Pritchard et al., 1975). Such results, taken together with the severe neurological symptoms that accompany the disease played a key role in showing the importance of purine salvage pathways in normal functioning of the brain.

Although ENT1 and ENT2 have been proposed as the transporters for Hx salvage, their respective K_m_ values for Hx are quite high (ENT1 K_m_ = 6.0 ± 0.5 mM; ENT2 K_m_ = 1.5 ± 0.2 mM) compared to typical plasma Hx concentrations (∼0.46 μM; see Section 2.8, below) (Yao et al., 2011), suggesting that some other more efficient mechanism existed for the transport of Hx across membranes. Thus, Bone et al. (2010, 2015) identified the existence of a nucleobase transporter in studies of Hx transport in cardiac microvascular endothelial cells (MVEC) (Bone and Hammond, 2007) and in MVEC from ENT1-/- mice). Although unable to identify the actual gene or protein involved, they termed the putative transporter as equilibrative nucleobase transporter 1 (ENBT1). That name was later used by Furukawa et al. (2015) who identified solute carrier family 43 member 3 (SLC43A3) as able to uptake nucleobases such as adenine, guanine, and hypoxanthine, but not nucleosides. Interestingly, they also showed that nucleobase import was greater when cells expressed both SLC43A3 and APRT (for adenine import) or HGPRT (for guanine import), suggesting that the nucleobase transporter and salvage enzyme work together in a cooperative fashion, with the final imported product being the nucleotides.

Interestingly, an earlier study by Wallgard et al. (2008) had looked for genes that were differentially expressed in microvascular endothelial cells, and they found *SLC43A3* differentially enriched in brain microvessels versus rest-of-brain tissue. In a review of FANTOM5, we found that all of twenty-one human endothelial cell samples expressed *SLC43A3*. As Furukawa had found that the *K*_m_ for Hx was 1.32 μM, which is close to reported normal Hx plasma concentrations, these results suggest that SLC43A3 functions in the microvasculature of various tissues to facilitate purine uptake and salvage. Interestingly, all FANTOM5 skeletal muscle samples also expressed the gene, suggesting a role for nucleobase import and salvage despite the contradictory results from research involving intense muscular exercise.

### Serum Urate Is a Biomarker of ATP Consumption

If that purine salvage process fails to salvage Hx back into high-energy phosphate molecules, and instead, XOR degrades it to X, then X can only be further degraded by XOR to UA (Figure 1). The degradation process may take place within cells if they express XOR or following excretion of Hx from a cell when it traverses organs with XOR activity such as the liver or endothelium. Consequently, processes maintaining cellular energy charge may degrade the molecular scaffold for ATP into a form (UA) that cannot be salvaged back into energy storing molecules. As described above, after degradation to UA, new purine molecules would need to be salvaged from dietary intake or resynthesized *de novo* at an energy expense of 7 high-energy phosphate bonds from 6 ATP molecules to regenerate IMP (Figure 1; Garrett and Grisham, 2016).

Previous results suggest that serum UA is a biomarker of ATP consumption, since rapid ATP consumption can lead to purine degradation and an increased rate of UA production in humans. For example, intense muscular exercise (Hellsten et al., 1999; Stathis et al., 1994, 1999), fructose challenge (Budillon et al., 1992; Donderski et al., 2015), alcohol intake (Lieber, 1965; Schmidt et al., 2013), and high brain activity (Salvadore et al., 2010; Goodman et al., 2016) can lead to energy crisis and resultant hyperuricemia. In addition, vascular regions undergoing ischemia-reperfusion may produce increased purine degradation products; a sign that a local energy crisis has occurred. For example, one study investigated the difference in the concentrations of adenine nucleotide degradation products between the aortic root (Ao) and the coronary sinus (Cs), before, during, and after cross-clamping of the aorta during cardiopulmonary bypass (Lazzarino et al., 1994). The authors found that pre-clamping Cs blood was slightly increased for Ado, Ino, Hx,and X (< 1 μmol/L), while UA was increased > 10 μmol/L, showing that XOR was present within the coronary vasculature. The Cs-Ao difference for purine degradation products increased 2–3 fold during the ischemic period, and decreased afterward. In addition, malondialdehyde, a marker of oxidative stress, significantly increased while the aorta was clamped, but there was no apparent correlation with post-operative outcomes, suggesting that oxidative stress in such pathologic situations may not be as important as is often thought.

In contrast, UA production is low in infirm aged people (Beberashvili et al., 2015, 2016), those with low nutrition (Beberashvili et al., 2016), and patients with certain neurodegenerative disorders (Rinaldi et al., 2003; de Lau et al., 2005). For multiple sclerosis (MS), decreased plasma/serum UA levels were observed in multiple studies (Druloviæ et al., 2001; Sotgiu et al., 2002; Liu et al., 2012; Moccia et al., 2015), but some studies have also observed that UA levels were increased compared to control subjects (Amorini et al., 2009; Tavazzi et al., 2011). Tavazzi et al. (2011) identified relapsing-remitting MS patients as having lower UA levels compared to secondary progressive or primary progressive MS patients, and the authors have described serum purine metabolite concentrations as related to an imbalance in energy production (Lazzarino et al., 2017).

### Inhibited Purine Degradation Maintains ATP Levels

Evidence suggests that inhibition of purine degradation leads to alterations of bioenergetic pathways and conserved ATP levels. Data that supports this conclusion is found in genetic deficiencies of the *AMPD* gene family, as well as from clinical and experimental analyses in various organ systems. AMPD enzymes catalyze the conversion of AMP to IMP (Figure 1), and expression of the three AMPD genes, *AMPD1, AMPD2*, and *AMPD3*, are differently expressed in various organs and in various types of cells (Morisaki et al., 1990; Mahnke-Zizelman and Sabina, 1992).

AMPD1 is very strongly expressed in skeletal muscle and diaphragm (Morisaki et al., 1990), and it has been examined extensively for the impact of a non-sense mutation that is common in European ancestry populations but rare in most other world-wide population samples (rs17602729; Gln45Ter; C34T; *AF*_T-EUR_ = 0.123 in 1000 Genomes)^5^ Originally, deficiency in AMPD1 due to rs17602729 homozygosity (TT) was identified as a putative cause of myopathy (Fishbein et al., 1978). However, greater than 1% of European ancestry populations are AMPD1 deficient, and many of those subjects are asymptomatic, which suggests a heterogeneity of its effects or that other factors might be involved (Verzijl et al., 1998; Sabina and Holmes, 2001; Hanisch et al., 2008). Studies of elite athletes found significantly less C34T carriers compared to controls, with more skewed genotype frequencies in sprint/power athletes versus endurance and mixed-event athletes, and there appeared to be a deficit of TT individuals (Rubio et al., 2005; Muniesa et al., 2010; Tsianos et al., 2010; Ciȩszczyk et al., 2011, 2012; Ginevičienė et al., 2014; Grealy et al., 2015). However, endurance athletes who had reached the elite level who were heterozygous appeared to generally perform comparably to CC individuals (Rubio et al., 2005; Tsianos et al., 2010). Among various exercise studies of normal and AMPD1 deficient subjects (Norman et al., 2001; Tarnopolsky et al., 2001; De Ruiter et al., 2002; Fischer et al., 2007; Norman et al., 2008), the study with the largest sample-size (n_CC_ = 89; n_CT_ = 38; n_TT_ = 12) (Fischer et al., 2007), which performed a short-term high-intensity Wingate cycling test, found that TT subjects exhibited decreased mean-power output and increased fatigue. However, compared to CC and CT individuals, they did not experience depletion of ATP at the end of the test (Norman et al., 2001). In heart failure patients, genetic AMPD1 deficient subjects exhibited decreased systolic blood pressure (de Groote et al., 2006; Safranow et al., 2009; Tousoulis et al., 2014; Feng et al., 2017), an increase in cardiac output (left ventricular ejection fraction) (Gastmann et al., 2004; Collins et al., 2006; Safranow et al., 2009; Feng et al., 2017), and improved heart failure prognosis (Loh et al., 1999; Gastmann et al., 2004). Interestingly, AMPD1 was also reported as a putative target of the diabetes drug metformin (Cheng et al., 2014), and AMPD1 deficient individuals have been reported to have decreased rates of diabetes (Safranow et al., 2009).

AMPD2 is most prominently expressed in non-skeletal muscle tissues, including brain, liver, and thymus (Morisaki et al., 1990). Genetic AMPD2 deficiency leads to increased ATP but a decrease of GTP and has been reported to lead to rare central nervous system (CNS) disorders (Akizu et al., 2013; Novarino et al., 2014).

AMPD3 is most strongly expressed in erythrocytes but is also found in other tissues, including skeletal muscle. Genetic AMPD3 deficiency has been observed, and it has been reported to lead to a 1.5 fold increase of ATP in erythrocytes without any health problem (Ogasawara et al., 1987; Yamada et al., 1994). Studies of AMPD3 deficient mice have also reported increased ATP levels (Cheng et al., 2012; Daniels et al., 2013; O’Brien et al., 2015).

In addition to genetic deficiencies of the AMPD isozymes, experimental studies have shown that perturbation of certain pathways can modulate cellular energy through AMPD. For instance, under conditions that mimic oxidative stress in studies of erythrocyte energy metabolism, Tavazzi et al. (2000, 2001) found that AMPD activity increased when incubated with H_2_O_2_ or NaNO_2_). Those studies suggest that changes to the local erythrocyte environment may result in deficient energy states due to purine loss. Another study by the same group investigated the effect of angiotensin II (AII) on matrix metalloproteinase activity (MMP) in the canine heart. AII administration increased MMP activity, as well as chamber diastolic stiffening and MMP activity, and decreased tissue bioenergetics, while treatment with an MMP inhibitor was shown to decrease the effects of AII and reduce purine loss, likely through inhibition of AMPD activity (Paolocci et al., 2006).

### XOR Inhibitor Is Beneficial for Diseases of Heart, Skeletal Muscle and Brain

As noted above, heart, skeletal muscle, and brain are among the organs requiring the largest amounts of energy and that are typical targets of mitochondrial diseases, and interestingly, recent human and animal studies have shown that XOR inhibitors (XOIs) such as allopurinol and febuxostat are useful for the treatment of disorders of heart, skeletal muscle and brain.

#### XOR Inhibitor Alone Is Good for the Heart

In animals, at least 11 papers reported that heart failure and ischemic heart disease were improved by the administration of allopurinol or febuxostat (DeWall et al., 1971; Arnold et al., 1980; Hopson et al., 1995; Pérez et al., 1998; Ekelund et al., 1999; Khatib et al., 2001; Ukai et al., 2001; Stull et al., 2004; Hou et al., 2006; Xu et al., 2008; Zhao et al., 2008; Yao and Seko, 2017; El-Bassossy et al., 2018). In humans, at least 20 papers, including randomized controlled trials (RCTs) and meta-analyses, reported that heart failure and ischemic heart disease were improved by the administration of allopurinol or febuxostat (Saugstad, 1996; Cappola et al., 2001; Gavin and Struthers, 2005; Pacher et al., 2006; Noman et al., 2010; Rentoukas et al., 2010; Thanassoulis et al., 2010; Kelkar et al., 2011; Gotsman et al., 2012; Hirsch et al., 2012; Agarwal and Messerli, 2013; Agarwal et al., 2013; Larsen et al., 2016; MacIsaac et al., 2016; Singh and Yu, 2016, 2017; Ansari-Ramandi et al., 2017; Foody et al., 2017; Singh et al., 2017; Bredemeier et al., 2018). Because of febuxostat’s more recent approval, most reports analyzed only allopurinol.

#### XOR Inhibitor Alone Is Good for Skeletal Muscle

Administration of allopurinol was reported to improve the effect of rehabilitation in the elderly (Beveridge et al., 2013), prevent muscle damage due to intense exercise such as playing soccer (Sanchis-Gomar et al., 2015) or crewing on an America’s Cup yacht (Barrios et al., 2011), and reduce muscle atrophy due to disuse (Ferrando et al., 2018). The Ferrando et al. (2018) study also confirmed an inhibitory effect of allopurinol on disuse muscle atrophy in experiments using mice. In addition to those studies, allopurinol has also been shown to reduce muscle fiber damage in a mouse model of chemically induced rhabdomyolysis (de Bragança et al., 2017) and increase maximal isometric force produced by muscles in aged mice (Ryan et al., 2011).

#### XOR Inhibitor Alone Is Good for the Brain

One hypothesis regarding the etiology of neurodegenerative disorders has been that UA has a protective effect. That was based on studies that showed that subjects with higher serum UA levels had lower hazard ratios (HR) or inverse correlation for PD (Alonso et al., 2007; Schwarzschild et al., 2008; Ascherio et al., 2009; Johansen et al., 2009; McFarland et al., 2013), AD (Maesaka et al., 1993; McFarland et al., 2013; Al-khateeb et al., 2015; Lu et al., 2016), amyotrophic lateral sclerosis (ALS) (Abraham and Drory, 2014), and multiple-sclerosis (MS) (Liu et al., 2012). Based on those observations, several clinical trials have been designed to test the effect of Ino supplementation in neurodegenerative disorders, with the expectation being that Ino degradation in the body would increase blood and cerebrospinal fluid (CSF) levels of UA to presumably more protective levels. Three trials examined its use in MS patients, and while one suggested some effect (Markowitz et al., 2009), the other two that had larger sample-sizes showed no benefit of the Ino treatment (Gonsette et al., 2010; Muñoz García et al., 2015). More recently, the SURE-PD study was initiated to investigate Ino treatment in PD patients (The Parkinson Study Group SURE-PD Investigators et al., 2014). SURE-PD has released a proof-of-principal report that shows that Ino increases UA levels and that it is generally tolerated, but the study showed contradictory efficacy results depending on the PD measure examined, and additionally, the study was not powered to determine efficacy of the treatment.

In contradiction to those studies, recent analyses of large clinical databases suggest that the hypothesis that UA exerts a protective effect against neurodegenerative disorders is mis-directed. First, a 2015 Taiwanese study by Hong et al. (2015) reported that gout patients have lower risk of dementia than controls. At first glance, that would agree with the concept that UA exerts a protective effect. However, their results also indicated that gout patients treated with UA lowering drugs had lower risks of both vascular and non-vascular dementias. Second, a more recent report by Singh and Cleveland (2018a) analyzed United States Medicare data to examine the incidence of dementia with respect to use of XOIs and reported a lower occurrence of dementia in individuals receiving either high-dose allopurinol or 40 mg/day febuxostat. That is, compared with allopurinol less than 200 mg/day, higher allopurinol doses (200 to 299 and at least 300 mg/day) and febuxostat 40 mg/day dose were each associated with lower HRs for dementia: 0.80 (95% CI 0.64, 0.98), 0.59 (95% CI 0.50, 0.71), and 0.64 (95% CI 0.47, 0.86), respectively. In a companion study, Singh and Cleveland (2018b) also reported that use of UA lowering drugs was not associated with an increase in the rate of dementia in older adults. which suggests that UA itself does not have a protective effect against dementia. In addition, experimental animal studies have also shown the beneficial effects of XOIs on neurodegenerative disorders. Kato et al. (2016) demonstrated that XOIs delayed the pathological process of ALS in model mice (Nishino et al., 2008a), and the same authors have also reported that XOIs delayed the pathological process of AD in model mice and inhibited development of senile plaques and neurofibrillary tangles (Nishino et al., 2016).

### Mechanism of the Beneficial Effects of XOR Inhibitors on Heart, Skeletal Muscle, and Brain

Within the various reports on XOIs′ beneficial effects, several hypotheses have been raised. Namely, the mechanism of action is likely related to: (1) UA reduction, (2) reactive oxygen species (ROS) suppression, or (3) ATP enhancement.

Evidence suggests that XOIs′ UA lowering effect is unlikely to be the mechanism of those beneficial effects. In animals such as mice and rats, serum UA levels are very low even without XOI treatment (Watanabe et al., 2014), and therefore, the benefits of XOR inhibition that were observed in animal models are unlikely to be due to its further lowering. Also, of importance to brain-related studies, CSF UA concentrations are very low compared to those in blood, with human CSF levels about 5% of those seen in plasma samples ([UA_CSF_]∼12.8 μM; [UA_Plasma_]∼250 μM) (Harkness, 1988; Jiménez et al., 1989b). Those low levels are related to two things. First, as introduced above, XOR expression in brain tissue is very low, with human and mice brains having 0.8% and 0.44% of levels seen in the highest XOR expressing tissues (Supplementary Tables S1–S4). Second, the blood-brain barrier (BBB) and blood-cerebral spinal fluid barrier have been reported to be only weakly permeable to UA (Jiménez et al., 1989a; Redzic et al., 2001). Considering that evidence, UA reduction in serum is unlikely to affect the brain.

Although many reports hypothesized that the suppression of ROS is the mechanism of the beneficial effects of XOIs, that possibility also appears quite unlikely, since while heart, skeletal muscle and brain are among the organs that consume the largest amounts of energy, they are also among those with the lowest amounts of XOR in humans (Figure 2A; Linder et al., 1999), with XOR activity mostly limited to the vascular endothelium of those tissues (Kelley, 2015). Also, recent reports have shown that inhibition of ROS by scavengers is often harmful because minimal levels of ROS are useful for signal transduction and cancer inhibition (Diebold and Chandel, 2016; Scialò et al., 2016; Quijano et al., 2016). The high ATP consumption that occurs in those organs should also coincide with high ROS production, since ROS is produced along with the consumption of oxygen. However, ROS are produced mostly in mitochondria, and based on a rough calculation of the daily ratio of moles UA excreted to moles O_2_ consumed (UA_g.excr_ = 0.75 g, UA_mole.excr_∼2.97 × 10^-3^ moles; 22 moles O_2_ per day) (Ng et al., 1984; Engelberg, 1996), oxygen consumption by mitochondria is at least 5,000 fold higher than that consumed by XOR. Thus, ROS related damage is more likely to stem from mitochondria than from XOR.

Therefore, most of the ROS produced would stem from mitochondria and thereby can never be suppressed by XOIs. Of course, this may suggest that mitochondria may be damaged by ROS and that that damage leads to ATP depletion. In fact, the association of ROS with various diseases including neurodegenerative disorders may be through the damage of mitochondria by excessive ROS. However, that does not support inhibition of ROS production as the reason for the beneficial effects of XOR inhibition that has been observed in previous studies.

Based on those observations, it is unlikely that ROS inhibition is the reason for XOIs′ beneficial effects. Rather, ATP enhancement through enhanced purine salvage is the more likely mechanism. Support for ATP enhancement as the mechanism can be found in experimental studies using *in vitro* and animal models as well as human clinical studies.

One such study in the early 2000s of allopurinol’s impact on mitochondrial function examined rat livers under ischemic/hypoxic conditions and found that decreases in ATP levels after ischemia/reperfusion were attenuated with allopurinol treatment (Jeon et al., 2001). Another study around the same time-period examined prepared rat hearts under hypoxic conditions, and the authors reported that both ATP and total adenine nucleotide pools were enhanced after allopurinol administration (Khatib et al., 2001). Later, a report on canines examined the impact of XOIs on cardiac bioenergetics under conditions simulating myocardial ischemia, with subjects examined at rest and then with/without XOI under either basal cardiac work (BCW) or high cardiac work (HCW) after catecholamine administration (Lee et al., 2011). Without XOI, the ratio of PCr/ATP and ADP levels were unchanged at BCW, but the PCr/ATP ratio decreased and levels of ADP increased at HCW. In contrast, with XOI administration, the ratio of PCr/ATP increased and levels of ADP decreased at BCW, while at HCW the increase in ADP levels was attenuated compared to no XOI. Similarly, a study of non-ischemic cardiomyopathy patients showed using ^31^P magnetic resonance spectroscopy (31P-MRS) that allopurinol increased the flux through creatine kinase (CK) and increased the rate of ATP synthesis as well as decreased ADP levels (Hirsch et al., 2012). In a neurological study of hypoxia/ischemia in piglets, authors reported that allopurinol pre-treatment attenuated a decrease in the PCr/Pi ratio, which is another measure of cellular energy status (Peeters-Scholte et al., 2003). Further, a study of potential causes of MS that used a mouse model of experimental autoimmune encephalomyelitis (EAE) reported that XO played a role in mechanisms leading to demyelination, and that EAE progression was reduced with XOI treatment (Honorat et al., 2013). A follow-up study examined NeuroA2 cells treated with rotenone, which causes dysfunction of mitochondrial electron transport and reduces ATP levels, and found that XOI administration to rotenone treated *in vitro* culture increased cellular ATP levels (Honorat et al., 2017).

### Hypoxanthine Is Important in Neurodegenerative Disorders

It has been reported that serum UA is reduced in patients with neurodegenerative disorders such as PD (Havelund et al., 2017; Sakuta et al., 2017; Wen et al., 2017), ALS (Abraham and Drory, 2014; Paganoni et al., 2018; Zhang et al., 2018), and AD (Euser et al., 2009; Lu et al., 2016), and as mentioned above, many researchers consider that these observations represent a direct protective role of UA suppressing ROS in the brain (Kori et al., 2016). However, since XOR is not expressed in the brain, and UA levels in the brain are naturally very low, it is more likely that low UA in neurodegenerative disorder patients reflects physiologic and biochemical changes that result in decreased energy production and low ATP consumption.

Such a hypothesis is supported by FDG-PET studies of the brain, which use the glucose analog FDG (fluoro-deoxy glucose) to assay metabolic activity through regional glucose uptake. That glucose uptake represents some glycolytic but predominantly mitochondrial activity in the brain for production of ATP (Mosconi, 2013). FDG-PET studies have identified increased regions of hypometabolism, and thus reduced ATP consumption, in the brains of patients with certain neurodegenerative diseases such as AD (Johnson et al., 2012; Lin and Rothman, 2014). Recently, a European task-force recommended use of FDG-PET in answering a number of clinical questions including the differential diagnosis of AD and PD from similar pathologies (Nobili et al., 2018).

If ATP consumption in the brain is reduced in such diseases, the UA precursor Hx should also be low. In line with that, a recent meta-analysis found that a number of metabolomic studies of PD patients identified decreases of compounds either related to energy production or purine-related pathways (Havelund et al., 2017). Among the latter, one report found that Hx levels in PD patients’ plasma were decreased by 41% while UA was decreased by 14% (Johansen et al., 2009). In addition to that data, a proteomics study targeting hundreds of proteins for incidental AD in the longitudinal Framingham Offspring Cohort identified elevations of anthranic acid and glutamic acid and reductions of Hx and taurine (Chouraki et al., 2017), and a MALDI-MSI analysis targeted at hundreds of proteins in Alzheimer’s model mice detected elevations of Hx and taurine (Esteve et al., 2017). Although those results are suggestive of a relationship between purine metabolism and the neurological diseases, we should also note that another potential cause of reduced Hx levels could be decreased physical activity that might accompany PD and AD.

It is of interest that both Hx and taurine were found among hundreds of compounds whose concentrations were different between disease and control subjects. However, in the mouse model, both Hx and taurine were increased, while they were decreased in human AD patients. This may reflect a difference as to the pathogenic mechanisms for AD between mice and humans, with a common facet of both being that unfolding and degradation of mis-folded/abnormal proteins such as amyloid or tau by proteasomes carries a high energy cost (Benaroudj et al., 2003; Peth et al., 2013). In the case of the mouse model, excessive production of misfolded proteins requires the expenditure of large amounts of ATP in order to degrade them, which is evidenced by increased purine degradation products such as Hx. In contrast, in humans the decreased levels of degradation products from energy production and purine pathways reflect energy production dysfunctions that result in a reduced capacity to degrade abnormal proteins. Thus, the elevation of Hx in the model mice and its reduction in the human patients can be explained by the different mechanisms by which amyloid beta and tau accumulation occur between model mice and human disease.

**FIGURE 3:**
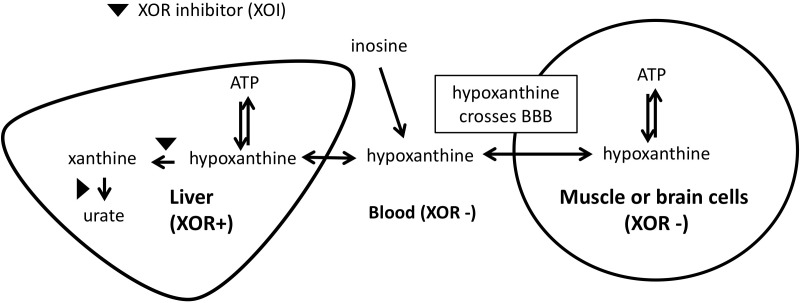
Mechanism of ATP enhancement by XOI or XOI+ inosine.

The mechanism by which we propose that XOIs enhance ATP is illustrated in Figure 3, which suggests that the blood concentration of Hx plays an important role. Since XOR exists mostly in liver in humans, the inhibition of XOR leads to the elevation of Hx in the liver, and eventually in the blood. As noted above, for each molecule of purine that is lost, seven high-energy phosphate bonds are needed for the *de novo* synthesis of IMP. Since elevated Hx saves purines for salvage, XOI should lead to the enhancement of ATP. Support for that was seen in animal models, such that when a XOI alone was administered to mice or rats, marked elevation of Hx occurred (Schmidt et al., 2009; Szasz et al., 2013; Kato et al., 2016). Similarly, in humans, allopurinol treatment of cancer patients before chemotherapy treatment was shown to increase Hx levels by about 1.5-fold (Wung and Howell, 1984).

With respect to brain levels of Hx, under normal physiologic conditions the mean concentration of Hx in CSF was between 1.8 and 2.0 μM from two reports (Harkness and Lund, 1983; Amorini et al., 2009). In blood, reported mean plasma/serum levels for Hx have varied widely as 0.46 μM (Wung and Howell, 1980), 1.5μM (Harkness and Lund, 1983), 4 μM (Amorini et al., 2009), and 32.4 μM (Psychogios et al., 2011), with the most representative value coming from the Wung and Howell (1980) report, which showed clearly that measured Hx concentrations in plasma/serum increases with time between blood draw and isolation of the plasma/serum. Based on those values, CSF in non-diseased individuals appears to be about three to 4-fold enriched for Hx compared to plasma. That enrichment within CSF (Harkness and Lund, 1983) presumably relates to the lack of XOR in brain. Since Hx is known to cross the BBB (Jiménez et al., 1989a), excess Hx produced in the CNS would be expected to move to the blood. However, with XOI treatment, blood levels of Hx should become elevated, and the BBB’s permeability to Hx should allow it to move into the CNS. Alternatively, the less favorable transport kinetics should reduce its movement from the CNS to the main circulation. An analogous situation was observed in a previous study of LND patients who are deficient for HGPRT, the enzyme that salvages Hx to IMP, and thus lack a key node of the purine salvage pathway (Figure 1; Jiménez et al., 1989a). Hx in HGPRT(-) patients was elevated 3.9-times and 6.4-times compared to their reported normal plasma and CSF, respectively, and Hx in HGPRT(-) patients’ CSF was 2.3-times more than in plasma ([Hx_Normal-CSF_]∼2.7 μM, [Hx_Normal-plasma_]∼1.9 μM; [Hx_HGPRT(-)-CSF_]∼17.2 μM; [Hx_HGPRT(-)-plasma_]∼7.3 μM). Interestingly, treatment with an XOI (Allopurinol) increased Hx in the plasma and CSF to roughly equal levels ([Hx_HGPRT(-)-Allop-CSF_]∼35.0 μM; [Hx_HGPRT(-)-Allop-plasma_]∼38.62 μM), suggesting that as Hx could not be degraded by the liver due to XOR inhibition, its levels built-up in the blood and finally equilibrated with those in CSF.

### Provision of a Hypoxanthine Source in Addition to an XOR Inhibitor May Further Enhance ATP

The reports described above have shown that XOIs can have a beneficial effect on various diseases, but it may be possible to enhance the effect by simultaneous administration of a Hx source. As noted above, when a XOI alone was administered to mice or rats, a marked elevation of Hx occurred, but mice and rats produce over 25 times more UA per unit body weight as compared to humans (Horiuchi et al., 1999; Hosoyamada et al., 2016). Those higher rates of UA production occur because of the higher rate of energy generation and ATP consumption in small versus large mammals. Due to the difference in purine flux within the body, the substantial elevation of Hx in humans that would be needed to increase purine salvage may not be achieved by only inhibiting XOR. Similarly, provision of additional purines alone would not be beneficial, since oral purines will be substantially broken down to UA in the digestive tract or during absorption. Therefore, treatment using a combination of an XOI with additional purines would be expected to increase purine salvage, decrease energy expenditures by reducing *de novo* purine synthesis, and achieve better enhancement of cellular ATP.

### Combination Treatment With Administration of an XOR Inhibitor and Inosine

To examine that last question, clinical studies were performed using healthy human subjects. Treatment with high-dose XOI alone produced only a slight increase in levels of Hx and ATP, while treatment with Ino alone did not increase Hx or ATP (Kamatani et al., 2017). In contrast, only the combination of an XOI and Ino was observed to substantially increase blood levels of Hx and ATP in the human subjects. In an additional study, it was observed that Ino alone could enhance ATP in human erythrocytes incubated in saline, likely due to the lack of XOR in erythrocytes (Kamatani et al., 2018).

Based on those results, Kamatani et al. (2019) performed a small clinical study of the combined use of an XOI and Ino was performed in two mitochondrial disease patients. After treatment, brain natriuretic peptide decreased by 31% for one patient with mitochondrial myopathy, and for the other patient, who had mitochondrial diabetes, insulinogenic index was raised 3.1-fold. In addition to that small study, the combination therapy is currently under evaluation in a mid-sized study of 30 PD patients.

Therapy combining an XOI and Ino is similar to the treatment of deficiency for carbamoyl-phosphate synthetase 2 (CAD). Through treatment with oral uridine, CNS symptoms were dramatically improved (Koch et al., 2017).

## Conclusion

Over many years of research, a number of different groups have examined the effects of XOI treatment on disorders of various organ systems, especially of the heart, skeletal muscles, and brain. With the combination drug, it is possible that the disease status of patients with many such disorders will be improved in the future.

## Author Contributions

TJ, HJ, and NK contributed to the conception of the article. TJ analyzed the external data. TJ and NK wrote the first draft of the manuscript. All authors contributed to manuscript revision, and read and approved the submitted version.

## Conflict of Interest Statement

This work was performed under the auspices of StaGen Co., Ltd. TJ and NK are employees of StaGen Co., Ltd. NK holds stock in and is Chairman of StaGen Co., Ltd., and is also a Director of the Tsukuba International Clinical Pharmacology Clinic. StaGen Co., Ltd., has received a Japanese patent and has pending patent applications in other jurisdictions for use of XOI+inosine as an ATP enhancement therapy. The remaining author declares that the research was conducted in the absence of any commercial or financial relationships that could be construed as a potential conflict of interest.

## Footnotes

1 Downloaded on 9/25/2018 from: https://gtexportal.org/home/gene/XDH

2 Downloaded on 1/7/2019 from the track “FANTOM5 CAGE phase 1and2 human tracks pooled filtered with 3 or more tags per library and rle normalized” for the human *XDH* promoter region from: http://fantom.gsc.riken.jp/zenbu/gLyphs/#config=ZCsN4JmAA9wiyJYsBEdfeC;loc=hg19::chr2:31637532..31637635+

3 Li et al. (2017) Supplementary Table S6.

4 Downloaded on 1/7/2019 from the track “FANTOM5 CAGE Phase 1 and 2 mouse tracks pooled filtered with 3 or more tags per library and rule normalized” for the mouse *Xdh* promoter region from: http://fantom.gsc.riken.jp/zenbu/gLyphs/#config=DmO_zYuHPxvu83HbOzew1C;loc=mm9::chr17:74299467..74299576+

5 https://gtexportal.org/home/gene/XDH
